# Effects of functional endoscopic sinus surgery on olfactory and trigeminal function in chronic rhinosinusitis with nasal polyps

**DOI:** 10.1038/s41598-026-58587-8

**Published:** 2026-07-07

**Authors:** Arianna Soncini, Kwangsu Kim, Fabian Herfort, Coralie Mignot, Antje Haehner, Thomas Hummel

**Affiliations:** 1 Smell and Taste Clinic, Department of Otorhinolaryngology, University of Dresden Medical School, Technische Universität Dresden, Fetscherstrasse 74, 01307 Dresden, Germany; 2 Department of Medicine and Surgery, University of Parma, via Gramsci 14, 43121 Parma, Italy

**Keywords:** Chronic rhinosinusitis, Olfactory disorder, Functional endoscopic sinus surgery, Event-related potentials, Time-frequency analysis, Diseases, Medical research, Neurology, Neuroscience

## Abstract

Chronic rhinosinusitis (CRS) with and without nasal polyps has a prevalence of around 10%, being one of the most common chronic diseases in Europe. It is characterized by a wide range of symptoms leading to a high individual, clinical and socioeconomic burden due to loss of productivity of individual patients and challenging therapeutic processes. This study aimed to investigate the effects of functional endoscopic sinus surgery (FESS) on olfactory function and associated quality of life of CRS patients, particularly regarding postoperative differences in neuronal processes. Using psychophysical and electrophysiological tools, the goal was to quantify postoperative changes to predict the neuronal functions related to olfactory improvements after FESS and ultimately provide better therapeutic recommendations in the future. Twenty-five CRS patients with nasal polyps aged from 30 to 64 years (CRS group) and 20 healthy controls aged from 23 to 54 years (control group) participated in this study. Both groups were tested twice (interval 3 to 6 months), with the first examination of the patients taking place a few days before surgery. Testing included questionnaires (medical history including self-ratings of olfactory function and nasal breathing, SNOT-20 questionnaire), psychophysical methods (Sniffin Sticks for olfactory function [TDI score], Peak Nasal Inspiratory Flow) and electrophysiological methods (olfactory and trigeminal event-related potentials). CRS group patients were also subjected to preoperative clinical examination and preoperative CT-scans. While the CRS group improved in terms of self-ratings of the sense of smell and TDI scores, as well as nasal breathing and quality of life, the control group did not show major longitudinal differences. In addition, electrophysiological measures suggested that trigeminal sensitivity decreased after surgery. CRS patients showed benefits from surgery in terms of olfaction, nasal breathing and quality of life, at least 3–6 months after the intervention. Interestingly, electrophysiological measurements suggested a postoperative decrease of trigeminal sensitivity.

**Supplementary Information:**

The online version contains supplementary material available at 10.1038/s41598-026-58587-8.

## Introduction

With a prevalence of 5–12%, Chronic Rhinosinusitis (CRS) is one of the most common chronic diseases in Europe, comparable in numbers to chronic obstructive pulmonary disease (COPD) and diabetes mellitus^1–3^. On an individual level, CRS leads to a significant reduction in quality of life, sleep quality and everyday productivity due to a variety of symptoms (including olfactory disturbance)^4^, whereby the level of suffering and the effects of CRS on the quality of life are comparable to other chronic diseases such as asthma, rheumatism, angina pectoris or heart failure^4–6^.

The most important group of drugs for CRS treatment are topical and systemic corticosteroids^2,4^. Biologics like dupilumab are reserved for special indications and are increasingly being used^2,3,7^. The standard procedure for the surgical treatment of CRS is functional endoscopic sinus surgery (FESS), which is indicated if there is a lack of response to conservative treatments^2,4,8,9^. At a societal level, the high direct therapeutic costs (in particular long courses of illness with recurrent operations) and indirect costs (in particular through lost working hours and reduced productivity) have a significant impact on socio-economic requirements^2,10–12^. Due to the high clinical and socioeconomic importance, CRS needs to be further investigated, to identify the most effective therapy for each patient.

Approximately 67–78% of people affected by CRS also present with an olfactory disorder^2,13^ which, according to current knowledge, is caused by a complex interplay of obstructive and inflammatory mechanisms influencing neuronal processes of the sense of smell^2,14^. CRS is commonly classified into CRS with nasal polyps (CRSwNP) and CRS without nasal polyps (CRSsNP). CRSwNP is predominantly associated with type 2 inflammation, whereas CRSsNP is generally associated with non-type 2 inflammatory patterns. Olfactory impairment has been described as a major symptom of CRSwNP, affecting approximately 83–91% of patients, and is more frequent and severe than in CRSsNP^15^. Furthermore, olfactory dysfunction has been reported to occur earlier and with greater severity in patients with eosinophilic infiltration, which is commonly associated with type 2 inflammation^16–18^.

Previous studies have shown that FESS usually leads to an improvement in disease-specific symptoms and health-related quality of life in patients affected by CRS. However, a significant improvement in subjective and objective olfactory performance is difficult to predict although responsiveness to systemic steroids seems to be a good parameter^16,17^. Improvement in olfactory function after FESS has been reported in approximately 25–80% of patients across various studies^18–26^ although reported outcomes vary according to how olfactory dysfunction is defined and how postoperative olfactory function is assessed. Differences in follow-up duration and the use of subjective versus objective olfactory measures may further contribute to this variability. Notably, several studies^20,21^ have reported greater olfactory improvement in patients with CRSwNP than in other CRS populations, suggesting that the reduction of inflammatory burden and restoration of airflow to the olfactory cleft may be particularly beneficial in these patients. Improvements in both the inflammatory and obstructive components in the pathogenesis of CRS are discussed as an explanation for the positive influence of FESS on chemosensory function, although the exact mechanism is not yet fully understood^2^. In addition, little is known about changes of nasal trigeminal function before and after surgery^27–29^ despite the fact that the trigeminal system constitutes a significant part of the perception of odors and it provides most of the sensory information about nasal airflow^30^.

This study focused therefore on the chemosensory abilities of CRSwNP patients before and after surgery. Special attention should be paid to changes of neuronal processes of olfactory structures. So far there is only one study investigating CRS-induced olfactory disturbances on an electrophysiological level^31^ suggesting that improvements in olfactory function after FESS are associated with a higher probability of occurrence of olfactory event-related potentials (ERPs) as well as altered ERP amplitudes and latencies.

## Materials and methods

### Participants

This prospective case-control study was conducted according to the guidelines of the Declaration of Helsinki of 2013 and was approved by the local ethics committee (ethics application BO-EK-126032020). Written informed consent was obtained from all subjects participating in the study. Participants affected by CRS (CRS group) were recruited at the Department of Otorhinolaryngology of a tertiary care university hospital during consultation hours; subjects of the control group were recruited via public notices. Patients were included based on a clinical diagnosis of CRSwNP confirmed by nasal endoscopy and physician assessment, including evaluation of medical history and clinical findings. A total of 45 participants were included in this study. The first group (25 participants; 23 men, 2 women) was composed by patients affected by CRSwNP, a condition frequently associated with olfactory dysfunction^15^, aged between 18 and 65 years at the time of the first examination already waiting for FESS. The control group (20 participants; 17 men, 3 women) was formed by subjects aged between 18 and 65 years at the time of the first examination and with normal olfactory function defined as a threshold, discrimination, and identification (TDI) score ≥ 30.75. Exclusion criteria for both groups were: (1) pregnancy or breastfeeding; (2) smoking (at least 5 cigarettes per week); (3) drug abuse. Moreover, patients were excluded from the CRS group if they presented with olfactory dysfunction not caused by CRS (e.g. caused by viral infections) and subjects were excluded from the control group if they presented with olfactory disorders or sinus disease.

### Procedures

Subjects were evaluated at two timepoints, each appointment lasted about 150 minutes. In the CRS group, the first appointment (baseline) took place a few days before surgery and the second appointment (follow-up) 3–6 months after surgery. In the control group, the second appointment (follow-up) took place 3–6 months after the first appointment. For the CRS group the first examination included an ENT preliminary examination (independent from the study) with evaluation of Lund-Mackay score (LMS^32^ and endoscopy. Following written informed consent to participate in the study, subjective olfaction (SOLF), subjective nasal flow (SNF) and disease-related quality of life, expressed with Sino-Nasal Outcome Test-20 German Adapted Version (SNOT-20 GAV), were collected. These were followed by psychophysical measurements of peak nasal inspiratory flow (PNIF) and olfactory function using the Sniffin’ Sticks TDI smell test. Finally, EEG was performed to record event-related potentials (ERP) and for subsequent time-frequency analysis (TFA). The same examination procedures were carried out at the follow-up appointment 3–6 months after surgery. None of the patients underwent structured olfactory training during the postoperative follow-up period. The control group was also examined according to the scheme described, but no ENT examinations and no surgery were performed.

### Measures

#### Medical history questionnaire

The first half of the medical history questionnaire asked for general information on age, height, gender, diagnostic indication to FESS, current or previous medical conditions, previous vaccinations against or infections with SARS-CoV-2, accidents, previous head surgery, allergies, regular medication, current alcohol and cigarette consumption and frequent contact with chemicals, dusts or gases. In addition, questions were asked about the presence of nasal polyps, analgesic intolerance and bronchial asthma (Samter triad)^33^. The second half of the questionnaire focused on olfaction, in particular the subjects were asked to evaluate their subjective olfactory ability using a verbal rating scale, in which one of eight response options had to be selected (from “very good” to “normal” to “no olfactory perception”). The possible presence of parosmia or phantosmia had to be indicated as well.

#### Subjective olfaction (SOLF)

Subjects were asked to express the subjective perception of olfaction using a visual analogue scale (VAS) where 0 indicated “no olfaction” and 10 indicated “extremely good olfaction”.

#### Subjective nasal flow (SNF)

Subjects were asked to express the subjective nasal breathing using a visual analogue scale (VAS) where 0 stood for “totally blocked” and 10 stood for “extremely good”.

#### Sino-nasal outcome test-20 German adapted version (SNOT-20 GAV)

This is a validated German-language questionnaire for assessing the quality of life of CRS patients^34^. It is an adapted and translated version of the Sino-Nasal Outcome Test-20^35^. It comprises a list of 20 symptoms from the four areas of nose, ear/face, sleep and mental well-being. Each symptom is rated by the subject on a combined verbal and numerical rating scale from 0 = “no problem” to 5 = “as bad as it can be”. The total SNOT-20 score was calculated as the sum of the ratings of all 20 items. Because each item is scored from 0 to 5, the total score ranges from 0 to 100 points. The greater the severity of the symptoms and thus the reduction in quality of life, the higher the score^34^. To date, no minimum clinically important difference (MCID) has been defined in the literature for the SNOT-20 score. Therefore, no MCID-based analysis was performed. Changes in SNOT-20 scores were evaluated using the difference between baseline and follow-up measurements and corresponding statistical comparisons.

#### Peak nasal inspiratory flow (PNIF)

It was measured using a PNIF meter (In-check™ nasal inspiratory flow meter, Clement Clarke International, Ltd., Harlow, UK), which consists of a breathing mask connected to a tube via a plug-in system and is placed over the closed mouth and nose of the subject examined to form a seal. During the test subjects are asked to perform a forced nasal inspiration (with mouth closed) after a complete expiration. The resulting suction moves a ring inside the tube at the end furthest from the mask towards the breathing mask. This ring comes to a standstill at the end of inhalation. The position of the ring after the breathing manoeuvre marks the value of the maximum inspiratory volume flow achieved, which can be read on a calibrated scale on the tube in Liters per minute (L/min)^36^.

#### Psychophysical olfactory testing

The psychophysical olfactory test was carried out using the Sniffin’ Sticks^37,38^. The test battery, consisting of a total of 112 olfactory pens, includes 3 subtests to test three aspects of subject’s olfactory ability: threshold (T), discrimination (D) and identification (I). Based on the sum of the scores obtained in each subtest, which can range from 1 to 48, subjects can be defined as anosmic (no olfactory ability, TDI < 16.50), hyposmic (TDI 16.50–30.50) or normosmic (TDI ≥ 30.75)^38^.

#### Electrophysiological examination

Olfactometric measurement of olfaction was carried out according to a method developed by Kobal^39,40^, in which odorants are applied intranasally using air-dilution olfactometry and the neurophysiological reactions are measured by recording an electroencephalogram and the ERP derived from it^41^. For selective olfactory stimulation, we used phenylethyl alcohol (PEA; 50%, v/v), and for selective trigeminal stimulation, we used carbon dioxide (CO_2_, 45%, v/v)^39^. Both stimuli were delivered by a computer-controlled olfactometer (OM6b, Burghart MT, Holm, Germany) at a flow of 7 L/min. They were embedded into a constant stream of warmed (37 °C) and humidified (80% relative humidity) air. Air was delivered to one of the nostrils through Teflon™ tubing (3 mm inner diameter). Each odorant was applied 40 times with a stimulus duration of 200 ms and with a varying interstimulus interval (ISI) of 18 ± 2 s to prevent the subject from habituating to the regular odor application, starting with the olfactory stimuli by PEA and concluding with the trigeminal stimuli by CO_2_ after an approximately 3-min break.

Recordings took place in a well-ventilated room with participants comfortably seated. During the EEG recording, the subjects breathed through the mouth, performed a computer game-like task to maintain attention (“tracking task”) and were visually shielded by a curtain and acoustically shielded by white noise through headphones^42^.

The subject’s skin was cleaned at the facial electrode contact points with a skin preparation paste (SkinPure: Nihon Kohden, Tokyo, Japan) and then disinfected. The 8 facial electrodes (BioSemi Ag-AgCl flat-type electrodes, BioSemi, Amsterdam, Netherlands) were then placed in the appropriate positions using electrode gel (SignaGel Electrode Gel, Parker Laboratories Inc., Fairfield, USA). Facial electrodes 1–4 were placed in the area of the nasal root and were used to record the activity of the olfactory bulb^43^. Facial electrodes 5 and 6 were placed at the lateral corners of the eyes and were used to record oculomotor activity. The facial electrodes 7 and 8 were placed over the mastoids and were used as reference electrodes. EEG data were re-referenced to the linked mastoids prior to ERP analysis. After measuring the head, the subject was fitted with the appropriate electrode cap (BioSemi Headcap, 64 channels, 10/20 layout, medium/large) and closed tightly with Velcro. The correct position of the cap was checked, the contact points were filled with gel and the 64 electrodes (BioSemi pin-type active electrodes) were connected according to the international 10–20 system^44^. Each recording started 500 ms before stimulus onset and continued for 2000 ms after the stimulation. Baseline correction was performed using the 500-ms prestimulus interval (− 500 to 0 ms). A bandpass of 0.1–30 Hz was used. Trials contaminated by eye blinks, eye movements, or excessive movement artifacts were identified by visual inspection and excluded from averaging by an experienced examiner. The amplitudes and latencies of peaks N1 and P2 were measured for each chemosensory ERP (csERP) for the electrode positions Pz, Cz and Fz according to the recommendations of the literature on csERP^45,46^. This resulted in the peak-to-peak amplitudes (N1P2) as a measure of the strength of the stimulus response.

After 40 stimulations (corresponding to a total duration of approximately 12 min for PEA and CO_2_ at 0.2 s stimulus duration and 18 ± 2 s ISI), each odorant was assessed by the subject in terms of subjective intensity (numerical analogue scale from 0 to 10, where “0 = no perception” and “10 = maximum intensity”) and pleasantness (numerical analogue scale from − 5 to + 5, where “− 5 = maximally unpleasant”, “0 = neutral” and “+5 = maximally pleasant”).

### Data analysis

The statistical analysis was carried out using IBM SPSS Statistics (Version 29.0 for Windows, SPSS Inc., Chicago, Illinois, USA) and Microsoft Excel (Version 2401 for Windows, Microsoft Corporation, Redmont, Washington, USA). The Shapiro–Wilk test was used to test for normal distribution of the parameters collected. Mann–Whitney U test for independent samples was used for non-normally distributed parameters to detect group differences. The Pearson Chi-square test was used to evaluate the distribution of sex in both groups. The course analysis of data within a group was carried out using the t test for dependent samples in the case of normal distribution and using the Wilcoxon test in the case of non-normal distribution. Effect sizes were defined using Cohen’s d as follows: |Cohen’s d| ≥ 0.2 → small effect; ≥ 0.5 → medium effect; ≥ 0.8 → strong effect (Cohen, 1988). The ERP success rates were analysed using the McNemar test with regard to a difference in progression within the respective groups. Metric parameters were given as mean values (± standard deviation; minimum-maximum) and absolute (relative) frequencies for categorical parameters. A repeated-measures analysis of variance (ANOVA) with age included as a covariate was used to compare the results of the two groups over time to control for its potential effect on the results. Given the significant age difference between the CRS and control groups, the influence of age was additionally evaluated for all subjective, psychophysical, chemosensory rating, and electrophysiological outcome measures. The homogeneity of regression slopes assumption was assessed by testing the Age × Group interaction. Correlations were performed for normal distribution according to Pearson and for non-normal distribution according to Spearman. The significance level was set at *p* < 0.05. Two-sided testing for significance was always used.

## Results

### Population characteristics

We included 45 participants, 25 belonged to the CRS group (23 men, 2 women) and 20 to the control group (17 men, 3 women). All 20 people in the control group took part in both appointments. Of the initial 25 CRS patients, 17 (68%) attended the second appointment. For the epidemiological characteristics of the population all patients attending the first appointment were considered, while for the rest of the analysis we considered only patients who attended also the follow-up. Mean age of the CRS group was 48 years (± 11; range 30–64 years) while the mean age of the control group was 32 years (± 7; range 23–54 years). The age difference between the two groups was found to be statistically significant (*p*<0.001). On the other hand, no gender distribution difference was observed between the two groups (*p*= 0.46), men were predominantly represented within both groups. Eighteen (72%) subjects of the CRS group had no history of sinus surgery, 5 (20%) had one previous surgery while 2 (8%) had at least 2 sinus surgeries in their medical history.

**Fig. 1:**
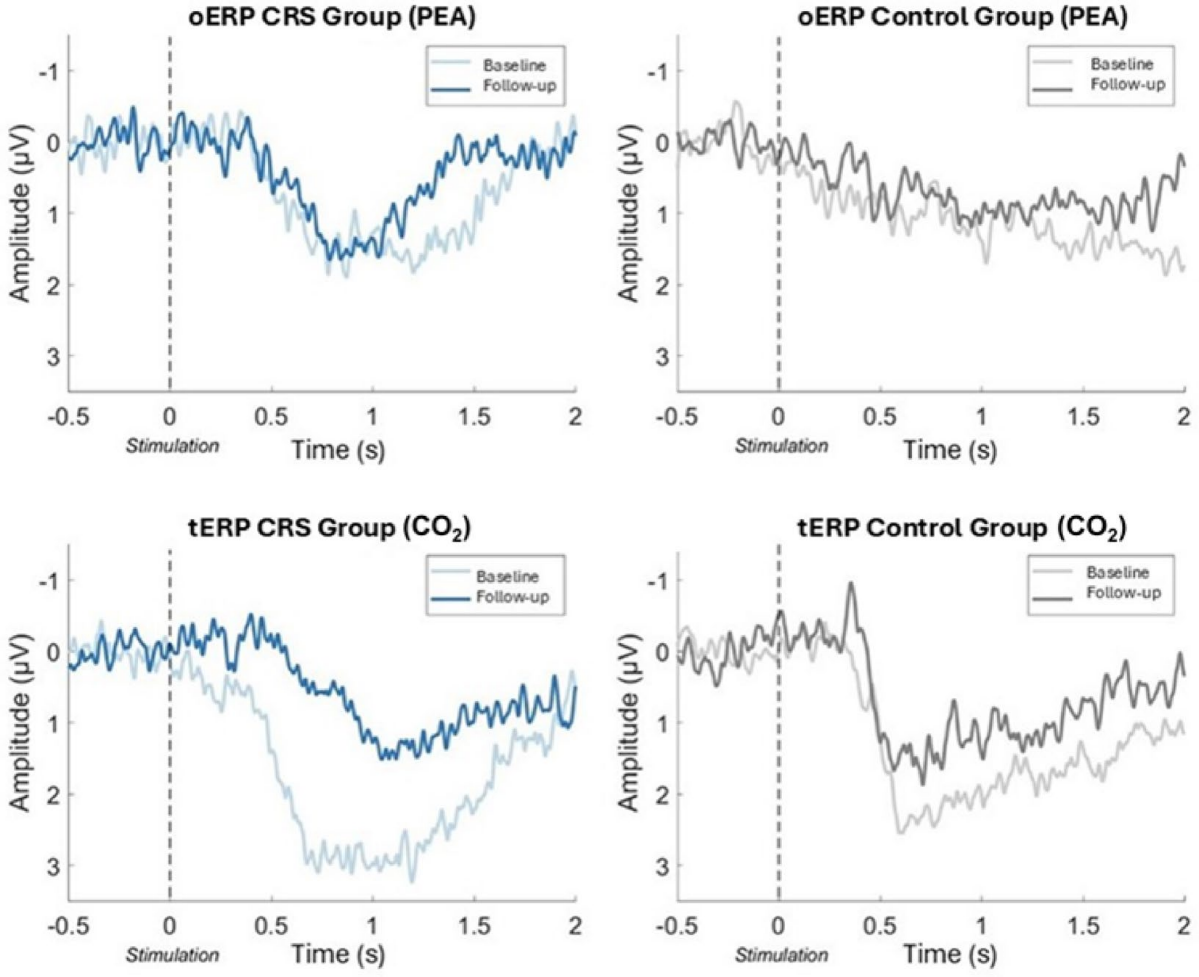
Grand Means of ERP. Averaged csERP of both examination times shown for the CRS group (left, blue) and the control group (right, gray) for PEA (oERP, top, for the Pz channel) and CO_2_ (tERP, bottom, for the Cz channel). The time point “0 s” represents stimulus onset. Both groups show larger tERP amplitudes at the baseline compared to the follow-up. In addition, tERP amplitudes N1P2 decreased for patients while they increased slightly for controls.

### Self-rated sensitivity parameters

#### CRS group

A significant improvement was observed from baseline to the follow-up after surgery in mean SOLF (t test: t[16] = − 3.07; *p* = 0.007; Cohen’s d = − 0.74) as well as in mean SNF (t test: t[16] = − 4.21; *p* < 0.001; Cohen’s d = − 1.02). SOLF improved in 12 out of 17 cases (71%), it decreased in 4 cases (24%) and in 1 case (6%) it was unchanged. SNF increased in 15 cases (88%), in 1 case (6%) it decreased and in 1 case (6%) it was stable. SNOT-20 also ameliorated with a mean improvement of 12.3 points. The difference between baseline and follow-up scores was significant (t[16] = 5.17; *p* < 0.001; Cohen’s d = 1.25) (Table 1).

#### Control group

No significant difference was observed between baseline and follow-up measures in the control group for all the parameters SOLF, SNF and SNOT-20. Values of the parameters are reported in Table 1.

**Table 1:** Subjective parameters of CRS group and control group.

	CRS group		Control group	
	Baseline	Follow-up	Baseline	Follow-up
SOLF value	3.2 (± 2.0; 0.0-6.9)	4.6 (± 1.6; 1.1–7.3)	5.8 (± 1.2; 4.1–7.9)	5.8 (± 1.3; 3.5-8.0)
SNF value	3.7 (± 1.9; 1.1–7.3)	6.6 (± 2.1; 2.7–9.5)	5.8 (± 1.9; 2.3–8.8)	5.8 (± 1.9; 2.3–8.1)
SNOT-20 value	26.8 (± 10.6; 8–45)	14.5 (± 9.1; 2–30)	5.5 (± 4.9; 0–20)	6.0 (± 5.9; 0–24)

#### Differences between the two groups

Repeated-measures ANOVA with age as a covariate revealed a difference between the two groups for SOLF (*p* < 0.001), and SNOT-20 (*p* < 0.001), indicating that the difference between the two groups was consistent over time. On the other hand, no difference was found between the two groups for SNF (*p* = 0.329). An interaction between “group” and “session” was found for SOLF (*p* = 0.028), SNF (*p* = 0.008) and SNOT-20 (*p* < 0.001), suggesting that the effect of the session varied depending on the group. Neither age nor the Age × Group interaction significantly influenced these outcomes (Supplementary Table S1).

### Psychophysical parameters of olfactory function and peak nasal inspiratory flow

#### CRS group

Results from psychophysical tests are summarized in Table 2. PNIF improved from baseline to follow-up, with a mean improvement of 36 L/min and the difference between the two timepoints was statistically significant (t test: t[16] = − 3.95; *p* = 0.001; Cohen’s d = − 0.91). In particular, PNIF increased after surgery in 14 out of 17 patients (82%) and it decreased in 3 cases (18%). TDI also increased with a significant difference between scores at baseline and at follow-up (t test: t[16] = − 3.93; *p* = 0.001; Cohen’s d = − 0.95), with an improvement of 6.5 point in mean TDI score and of 2 points in mean threshold, discrimination and identification values (Table 2).

In addition to the increase in mean TDI scores, changes in olfactory function categories were evaluated. Before surgery, 5 patients were classified as anosmic, 6 as hyposmic, and 6 as normosmic. At follow-up, 3 of the 5 anosmic patients (60.0%) improved to a higher olfactory category, including 1 patient who reached normosmia. Moreover, 2 of the 6 hyposmic patients (33.3%) improved to normosmia. Overall, 3 of 11 patients (27.3%) with preoperative anosmia or hyposmia achieved normosmic olfactory function after surgery.

#### Control group

As shown in Table 2, psychophysical parameters of olfactory function were stable between the two timepoints and the only difference observed between baseline and follow-up was for the discrimination ability, which decreased with time (t test: t[19] = 2.48; *p* = 0.023; Cohen’s d = 0.56).

**Table 2:** Psychophysical parameters of CRS group and Control group.

	CRS group		Control group	
	Baseline	Follow-up	Baseline	Follow-up
PNIF (L/min)	116 (± 57; 40–210)	152 (± 43; 90–230)	176 (± 66; 50–280)	182 (± 55; 90–310)
TDI score	21.5 (± 10.5; 7.0–34.5)	27.0 (± 9.5; 7.0–39.0)	34.5 (± 3.0; 31.0–40.3)	34.2 (± 3.0; 28.5–40.3)
Threshold	3.0 (± 2.3; 1.0–8.5)	4.9 (± 3.6; 1.00–12.00)	6.7 (± 2.2; 3.50–10.50)	6.7 (± 2.2; 2.50–10.3)
Discrimination	8.9 (± 4.4; 3–14)	10.6 (± 3.3; 5–14)	13.9 (± 1.1; 12–16)	13.3 (± 1.5; 10–16)
Identification	9.5 (± 4.8; 2–16)	11.7 (± 3.3; 4–15)	13.8 (± 1.7; 11–16)	14.3 (± 1.5; 11–16)

#### Differences between the two groups

Repeated-measures ANOVA with age as a covariate revealed a difference between the CRS and control groups for both PNIF (*p* = 0.034), and TDI (*p* = 0.011). Moreover, analyzing the subsets of TDI, a difference was found for threshold (*p* = 0.019) and discrimination (*p* = 0.005) while no significant difference was observed for identification. The interaction between “group” and “session” was not significant for PNIF, discrimination and identification but it was significant for TDI (*p* = 0.003) and threshold (*p* = 0.03). Neither age nor the Age × Group interaction significantly influenced these outcomes (Supplementary Table S1).

### Electrophysiological parameters

The mean peak-to-peak amplitudes (N1P2 amplitude), latencies (P1 latency) and duration of responses (P1P2) of both groups are summarized in Table 3.

#### CRS group

EEG datasets were only analyzed when available both at baseline and follow-up (not all datasets could be analyzed due to excessive artifacts or other technical issues): Paired datasets were available from 16 patients for PEA and 14 patients for CO_2_. Of these, preoperatively, olfactory ERP (oERP) could be derived in 13 of 16 cases (81%), trigeminal ERP (tERP) in 11 of 14 cases (79%). Postoperatively, oERP were detected in 14 of 16 cases (88%), tERP in 13 of 14 cases (93%) (Fig. 1).

The N1P2 amplitude in response to PEA increased in 4 of 11 cases (36%) and decreased in 4 cases (36%) postoperatively. These analyses were restricted to the 11 patients with detectable oERP responses at both baseline and follow-up. In 3 cases (27%) there was no difference. There were no significant differences between baseline and follow-up in N1P2 amplitude (t test: t[10] = 0.60; *p* = 0.56), and N1 and P2 latencies (t test: *p* = 0.37 and *p* = 0.33).

Mean N1P2 amplitude in response to CO_2_ decreased from baseline to the follow-up (t test: t[10] = 2.54; *p* = 0.03; Cohen’s d = 0.77). There were no significant differences for the N1 and P2 latencies (t test: *p* = 0.45 and *p* = 0.31) to CO_2_.

#### Control group

Datasets were available for both pre- and postoperative sessions for 20 recordings in response to PEA and 18 in response to CO_2_. On the first appointment, oERP could be derived in 19 out of 20 cases (95%), tERP in 17 out of 18 cases (94%). On the second session, oERP were detected in 20 out of 20 cases (100%), tERP in 17 out of 18 cases (94%) (Fig. 1).

There were no significant differences within the control group for the N1P2 amplitude (*p* = 0.62), and N1 and P2 latencies for PEA (*p* = 0.37 and 0.63). Moreover, there were no significant differences for the N1P2 amplitude (*p* = 0.73), and N1 and P2 latencies (*p* = 0.16 and 0.44) to CO_2_.

When comparing patients and controls, repeated-measures ANOVA with age as a covariate revealed a different pattern only for N1P2 amplitudes in response to stimulation with CO_2_: amplitudes decreased for patients whereas amplitudes increased for controls slightly (interaction between “group” and “session”: *p* = 0.032). Neither age nor the Age × Group interaction significantly influenced these outcomes (Supplementary Table S1).

**Table 3:** Amplitudes, latencies and duration of the responses of the CRS group and Control group for PEA and CO_2_ at recording position Cz.

	CRS group		Control group	
	Baseline	Follow-up	Baseline	Follow-up
*N1P2 amplitude (µV)*				
PEA	2.7 (± 1.5; 1.5–5.8)	2.8 (± 1.3; 1.3–5.7)	3.5 (± 1.9; 1.0-7.4)	3.1 (± 1.2; 1.3-7.0)
CO_2_	3.8 (± 1.8; 1.3–6.5)	2.6 (± 1.6; 1.2–7.1)	5.1 (± 2.7; 1.7–11.7)	5.5 (± 3.3; 1.6–12.4)
*N1 latency time (ms)*				
PEA	374 (± 85; 250–525)	392 (± 113; 232–643)	370 (± 82; 238–576)	402 (± 115; 301–699)
CO_2_	403 (± 100; 260–607)	404 (± 119; 270–676)	366 (± 88; 221–553)	416 (± 81; 293–617)
*P2 latency time (ms)*				
PEA	579 (± 97; 410–758)	621 (± 120; 439–779)	591 (± 121; 416–814)	607 (± 123; 424–813)
CO_2_	635 (± 141; 430–877)	622 (± 120; 410–795)	624 (± 94; 428–775)	654 (± 108; 477–816)

No significant difference was found between CRS and control groups in the progression from baseline to follow-up of ERP amplitudes, latency times. However, in patients there was a significant decrease in ERP N1P2 amplitudes in response to stimulation with CO_2_ which was the opposite for controls (interaction between “group” and “session”: *p* = 0.032).

### Subjective intensity and pleasantness

#### CRS group

The averaged intensity (numerical analogue scale from 0 to 10) and pleasantness (numerical analogue scale from − 5 to + 5) ratings of the test subjects are summarized in Table 4. The intensity of PEA increased by an average of 2.6 points postoperatively and the difference was statistically significant (*p* = 0.01). There were no significant differences for the rated intensity of CO_2_ between baseline and follow-up (*p* = 0.055) and neither for PEA (*p* = 0.24) and CO_2_ (*p* = 0.88) pleasantness.

#### Control group

In the control group no significant difference was found between baseline and follow-up in the subjective intensity (*p* = 0.372) and pleasantness of PEA (*p* = 0.666). Moreover, no difference was observed for the subjective intensity (*p* = 0.613) and pleasantness (*p* = 0.383) of CO_2_.

**Table 4:** Subjective intensity and pleasantness of the odorants in the CRS group and Control group for PEA and CO_2_.

	CRS group		Control group	
	Baseline	Follow-up	Baseline	Follow-up
*Intensity (0–10)*				
PEA	2.2 (± 2.2; 0–7)	4.8 (± 2.7; 0–9)	5.7 (± 2.4; 1–10)	5.3 (± 1.9; 3–9)
CO_2_	3.7 (± 2.9; 0–8)	5.0 (± 2.4; 0–9)	6.7 (± 2.1; 2–10)	6.5 (± 1.4; 4–9)
*Pleasantness (−5 to +5)*				
PEA	+ 0.4 (± 0.9; − 1 to +2)	+ 1.0 (± 1.8; − 3 to +5)	0.1 (± 1.9; − 3 to +4)	− 0.1 (± 1.9; − 3 to +4)
CO_2_	− 0.5 (± 1.7; − 4 to +2)	− 0.6 (± 1.3; − 3 to +2)	− 2.4 (± 1.4; − 5 to +1)	− 2.6 (± 1.0; − 4 - − 1)

#### Differences between the two groups

Repeated-measures ANOVA with age as a covariate revealed a difference between the CRS and control groups for PEA intensity (*p* = 0.004), CO_2_ intensity (*p* = 0.002) and CO_2_ pleasantness (*p* < 0.001). The interaction between “group” and “session” was significant only for PEA intensity (*p* = 0.004). Neither age nor the Age × Group interaction significantly influenced these outcomes (Supplementary Table S1).

### Correlations

SOLF improvement appeared to be correlated with improvements in SNOT (*r* = 0.39, *p* = 0.017), with improvements in SNF (*r* = 0.48, *p* = 0.003) and also with improvements in TDI score (*r* = 0.43, *p* = 0.008). SNOT improvement was also positively correlated with TDI score improvements (*r* = 0.47, *p* = 0.003) and SNF improvement (*r* = 0.47, *p* = 0.003). No significant correlations were found between PNIF improvement and SNOT improvement or SNF improvement. In addition, a preoperatively higher SNOT-20 score was associated with a greater SNOT-20 improvement (*r* = 0.60, *p* = 0.010).

## Discussion

To date, only a few studies have investigated CRS-induced olfactory disorders from an electrophysiological point of view. The focus of this prospective case-control longitudinal study was to investigate the chemosensory abilities of CRS patients a few days before and 3–6 months after sinus surgery, especially regarding postoperative changes in the neuronal processes of olfactory structures and to compare the results to healthy subjects.

The characteristics of the CRS group, including disease severity and comorbidities as well as the subjective and psychophysical improvements after sinus surgery, are consistent with the data from other studies in this field in recent years^8,47–50^. Consistent with previous studies, patients showed a significant improvement in SNOT-20 scores following surgery, indicating an overall improvement in disease-specific quality of life. The mean reduction in SNOT-20 scores observed in the present study was comparable to the improvements reported in larger cohorts following functional endoscopic sinus surgery^18,47,51,52^. The average SNOT-20 improvement of 12.3 points in this study also matches the SNOT-22 postoperative improvements of 12–25 points on average, described in the literature^18^. Also concordant with previous studies^18,51,53,54^ is the observation that a higher preoperative SNOT score is expected to result in greater postoperative improvement than less pronounced impairments. In their prospective cohort study from 2023, Hernandez and colleagues described that patients with more severe olfactory impairment could benefit more from endoscopic sinus surgery than those with less severe disease^55^, which is also observed in the present study. The subjective postoperative improvements are also largely consistent with the previously mentioned studies. In our population, the subjective improvements in nasal breathing were greater than the subjective improvements in olfaction and may have made the greater contribution to the improvement in quality of life.

With regard to TDI improvements after FESS, the results in the literature are generally more inconsistent: while some studies have observed postoperative olfactory improvements in just less than 25% of CRS patients^23^ when using psychophysical tests of olfactory function, larger reviews by Kohli and colleagues from 2016 and Alanin and colleagues from 2020 report postoperative olfactory improvements in 25–100% of cases^18,20^ although often self-ratings have been employed to gauge the success of surgery which can be misleading^56^. TDI improved in this study by more than 5.5 points in 47% of cases with no individualized deteriorations of more than 5.5 points: these results are within the average expected range of postoperative olfactory improvements. In 18% of cases, there was a postoperative reduction in the TDI value of less than 5.5 points compared to the preoperative examination. Causes for postoperative deterioration of olfactory function could be intraoperative injury to the olfactory epithelium, mucosal scarring during major polyp removal, persistent inflammatory and obstructive processes after surgery, particularly in the absence of postoperative application of a topical corticosteroid, and early recurrences of the disease as early as 3 to 6 months after surgery. In addition, undiagnosed viral infections could have led to olfactory disorders.

Several mechanisms may contribute to the postoperative improvement of olfactory function observed after FESS in patients with CRSwNP. First, removal of nasal polyps and restoration of sinonasal ventilation may improve the access of odorants to the olfactory cleft, thereby enhancing odor perception^18,20^. Second, surgery may reduce the local inflammatory burden within the sinonasal mucosa, particularly in patients with type 2 inflammation and eosinophilic infiltration^2,16^, both of which have been associated with olfactory dysfunction. Therefore, the observed improvement in TDI scores is likely the result of both conductive and inflammatory mechanisms.

Limited data are available in the literature about the electrophysiological changes after surgery. Previous studies suggested that olfactory performance enhancement after surgery could lead to a higher probability of ERP occurrence as well as larger peak-to-peak amplitudes and shorter latency times^31,57–62^. However, the presently observed high ERP success rates of around 80% before and 90% after surgery in the CRS group in particular deviates significantly from previous studies: Rombaux and colleagues were able to detect oERP in approximately one third of the tested patients with olfactory dysfunction^60^; Hu and colleagues also reported oERP success rates of approximately 30% in CRSwNP patients^31^. Hu and colleagues, also like the present study, found no significant correlation between psychophysical olfactory testing and the amplitudes and latencies of ERP in patients with CRSwNP with similar absolute values in pre- and postoperative comparisons. Similar absolute amplitudes and latencies were measured by Zhang and colleagues, although their data showed a strong negative correlation between olfactory threshold and ERP latency^62^, which was not the case in the present study. Brämerson and colleagues^57^ described significantly higher amplitudes in their publication from 2008, but using 0.3% butanol as the olfactory stimulant instead of PEA 50% v/v as in the present study.

Concerning trigeminal sensitivity, a significant decrease in ERP N1P2 amplitudes in response to stimulation with CO_2_ was observed, while controls showed a slight increase. To date, no studies have been published on effects of FESS on central-nervous processing of trigeminal input using electrophysiological measures. Poletti and colleagues investigated the effect of FESS on endonasal trigeminal detection threshold. Interestingly, a significant improvement of local trigeminal sensitivity was observed at the nasal septum after surgery, while on the lateral nasal wall and middle turbinate the threshold was not changed^63^.

The reason for the lower amplitudes of CO_2_ and lower power of PEA and CO_2_ in the CRS group could be biased by the test subjects at the second appointment. Experience shows that patients attend follow-up appointments especially if treatment is not successful. However, this could not be confirmed in our study. Another possible explanation for lower postoperative trigeminal response could be the surgical intraoperative damage to the trigeminal nerve endings present in the nasal mucosa. Such damage may happen more often than clinically recognized. In fact, a decreased trigeminal sensitivity has also been detected (while it was not actively reported) in patients following monopolar cautery of the sphenopalatine artery in proximity to branches of the trigeminal nerve for nasal bleeds^64^.

A longer follow-up could possibly reveal better outcomes, by giving more time to nerve fibers to recover.

In 2021, Whitcroft and colleagues published the results of their longitudinal study with 24 CRS patients and 17 healthy controls, which for the first time demonstrated both functional and structural neuroplasticity of central olfactory structures in relation to postoperative olfactory improvements 3 months after FESS in CRS^65^. A previous study already showed structural plasticity of olfactory cortical areas^66^. Interestingly, the 2021 publication reported an increase in the functional activity of olfactory networks, while at the same time certain olfactory areas showed a reduced gray matter volume postoperatively. However, previous work has shown correlations between improved olfactory function and increased gray matter volume of olfactory networks^65^. Still, in the present study, neuroplasticity of olfactory networks could not be clearly demonstrated by electrophysiological measurements, at least not under the assumption that postoperative functional improvements can be quantified by larger amplitudes or power.

Another aspect not previously discussed is the growing evidence regarding the use of structured olfactory training to regain olfactory skills. In 2020, Liu and colleagues published the largest cohort study to date with over 600 participants, including norm-, hyp- and anosmic individuals, who underwent structured olfactory training twice daily for an average of 31 weeks, compared with a control group without olfactory training^67^: olfactory training significantly improved olfactory performance in both patients and healthy individuals in approximately one third of cases, of a similar magnitude to the olfactory improvements after FESS in CRS patients. It should be noted that the regression analysis by Liu and colleagues did not include CRS patients, but mainly patients with post-infectious, post-traumatic and idiopathic olfactory disorders. The authors were able to demonstrate that olfactory training is more effective than no olfactory training and should therefore be considered as treatment for olfactory dysfunctions.

Several limitations of the present study should be acknowledged. First, the sample size available for some electrophysiological analyses was relatively small. A post-hoc sample size estimation based on the main ERP finding (N1P2 amplitude with CO_2_; Cohen’s dz = 0.77) indicated that approximately 16 paired datasets would be required to achieve 80% statistical power, whereas only 11 analyzable paired datasets were available in the present study. Therefore, the electrophysiological findings should be interpreted cautiously and warrant replication in larger cohorts. Second, some patients received concomitant medical treatment, including intranasal glucocorticoid spray therapy, according to clinical indications. Consequently, the independent effects of surgery and medical treatment on postoperative olfactory outcomes cannot be completely disentangled. In contrast, none of the patients underwent structured olfactory training during the follow-up period. Finally, the order of chemosensory stimulation was fixed, with PEA always preceding CO_2_. Although this approach was chosen to minimize potential carry-over effects of trigeminal stimulation on olfactory measurements and to reduce participant discomfort, an order effect cannot be excluded. Future studies may consider employing randomized stimulus presentation orders and standardized postoperative treatment protocols.

## Conclusions

In summary, the present study showed significant improvements in subjective and objective olfactory function, nasal breathing and the associated noticeable improvements in the patients’ quality of life 3–6 months postoperatively. Interestingly, these improvements of olfactory function could not be obtained using electrophysiological methods. However, ERP recordings suggested a decreased trigeminal sensitivity. These findings require further confirmation in future studies.

## Supplementary Information

Below is the link to the electronic supplementary material.

Supplementary Material 1

## Data Availability

The anonymized data are available from the corresponding author upon reasonable request.

## References

[1] Cuevas, M. & Zahnert, T. Chronic rhinosinusitis. *Laryngorhinootologie* **94**, 395–414 (2015). quiz 415–417. 10.1055/s-0035-1549891 26039039

[2] Fokkens, W. J. et al. European position paper on rhinosinusitis and nasal polyps 2020. *Rhinology* **58**, 1–464 (2020). 32077450 10.4193/Rhin20.600

[3] Marin, C., Hummel, T., Liu, Z. & Mullol, J. Chronic Rhinosinusitis and COVID-19. *J. Allergy Clin. Immunol. Pract.* **10**, 1423–1432 (2022). 35307579 10.1016/j.jaip.2022.03.003 PMC8926942

[4] Stuck, B. A. et al. Guideline for ‘rhinosinusitis’-long version: S2k guideline of the German College of General Practitioners and Family Physicians and the German Society for Oto-Rhino-Laryngology, Head and Neck Surgery. *HNO* **66**, 38–74 (2018). 28861645 10.1007/s00106-017-0401-5

[5] Gliklich, R. E. & Metson, R. The health impact of chronic sinusitis in patients seeking otolaryngologic care. *Otolaryngol. Head Neck Surg.* **113**, 104–109 (1995). 7603703 10.1016/S0194-59989570152-4

[6] Macdonald, K. I., McNally, J. D. & Massoud, E. The health and resource utilization of Canadians with chronic rhinosinusitis. *Laryngoscope* **119**, 184–189 (2009). 19117310 10.1002/lary.20034

[7] Pfaar, O. et al. Therapie der chronischen Rhinosinusitis mit Polyposis nasi (CRScNP) mit monoklonalen Antikörpern (Biologika): S2k-Leitlinie der Deutschen Gesellschaft für Hals-Nasen-Ohren-Heilkunde, Kopf- und Hals-Chirurgie (DGHNO-KHC) und der Deutschen Gesellschaft für Allgemeinmedizin und Familienmedizin (DEGAM). *HNO* **71**, 256–263 (2023). 36941387 10.1007/s00106-023-01273-2 PMC10066152

[8] Bitter, T. & Guntinas-Lichius, O. Funktionelle endoskopische ­Nasennebenhöhlenchirurgie (FESS). (2019). 10.1055/a-0830-3960 31167294

[9] Weber, R. Aktueller Stand der endonasalen Nasennebenhöhlenchirurgie. *Laryngorhinootology* **94**, S64–S142 (2015). 10.1055/s-0035-1545353 25860497

[10] Leland, E. M., Zhang, Z., Kelly, K. M. & Ramanathan, M. Role of environmental air pollution in chronic rhinosinusitis. *Curr. Allergy Asthma Rep.* **21**, 42 (2021). 34499234 10.1007/s11882-021-01019-6 PMC8590820

[11] Rudmik, L. et al. Productivity costs in patients with refractory chronic rhinosinusitis: Productivity costs in patients with refractory CRS. *Laryngoscope* **124**, 2007–2012 (2014). 24619604 10.1002/lary.24630 PMC4125547

[12] Smith, K. A., Orlandi, R. R. & Rudmik, L. Cost of adult chronic rhinosinusitis: A systematic review. *Laryngoscope* **125**, 1547–1556 (2015). 25640115 10.1002/lary.25180

[13] Kohli, P. et al. The prevalence of olfactory dysfunction in chronic rhinosinusitis. *Laryngoscope* **127**, 309–320 (2017). 27873345 10.1002/lary.26316 PMC5258829

[14] Jafek, B. W. Biopsies of human olfactory epithelium. *Chem. Senses*. **27**, 623–628 (2002). 12200342 10.1093/chemse/27.7.623

[15] Soler, Z. M. et al. Reduced sense of smell in patients with severe chronic rhinosinusitis and its implications for diagnosis and management: A narrative review. *Adv. Ther.* **41**, 4384–4395 (2024). 39382822 10.1007/s12325-024-02984-w PMC11550237

[16] Bogdanov, V., Walliczek-Dworschak, U., Whitcroft, K. L., Landis, B. N. & Hummel, T. Response to glucocorticosteroids predicts olfactory outcome after ESS in chronic rhinosinusitis. *Laryngoscope* **130**, 1616–1621 (2020). 31373696 10.1002/lary.28233

[17] Otten, J. J. et al. Steroid responsiveness predicts olfactory function recovery in dupilumab treated CRSwNP. *Rhinology* **62**, 403–409 (2024). 38775362 10.4193/Rhin23.452

[18] Alanin, M. C. & Hopkins, C. Effect of functional endoscopic sinus surgery on outcomes in chronic rhinosinusitis. *Curr. Allergy Asthma Rep.* **20**, 27 (2020). 32462321 10.1007/s11882-020-00932-6

[19] Delank, K. W. & Stoll, W. Olfactory function after functional endoscopic sinus surgery for chronic sinusitis. *Rhinology* **36**, 15–19 (1998). 9569436

[20] Kohli, P. et al. Olfactory Outcomes after Endoscopic Sinus Surgery for Chronic Rhinosinusitis: A Meta-analysis. *Otolaryngol. Head Neck Surg.* **155**, 936–948 (2016). 27576679 10.1177/0194599816664879

[21] Lind, H., Joergensen, G., Lange, B., Svendstrup, F. & Kjeldsen, A. D. Efficacy of ESS in chronic rhinosinusitis with and without nasal polyposis: a Danish cohort study. *Eur. Arch. Otorhinolaryngol.* **273**, 911–919 (2016). 26031891 10.1007/s00405-015-3667-9

[22] Litvack, J. R., Mace, J. & Smith, T. L. Does olfactory function improve after endoscopic sinus surgery? *Otolaryngol. Head Neck Surg.* **140**, 312–319 (2009). 19248934 10.1016/j.otohns.2008.12.006 PMC2668517

[23] Pade, J. & Hummel, T. Olfactory function following nasal surgery. *Laryngoscope* **118**, 1260–1264 (2008). 18438263 10.1097/MLG.0b013e318170b5cb

[24] Paksoy, Z. B., Cayonu, M., Yucel, C. & Turhan, T. The treatment efficacy of nasal polyposis on olfactory functions, clinical scoring systems and inflammation markers. *Eur. Arch. Otorhinolaryngol.* **276**, 3367–3372 (2019). 31473779 10.1007/s00405-019-05619-x

[25] Saedi, B., Sadeghi, M., Yazdani, N. & Afshari, A. Effectiveness of FESS in smell improvement of sinusitis patients. *Indian J. Otolaryngol. Head Neck Surg.* **65**, 283–287 (2013). 24427662 10.1007/s12070-011-0439-8 PMC3738785

[26] Yan, X., Whitcroft, K. L. & Hummel, T. Olfaction: Sensitive indicator of inflammatory burden in chronic rhinosinusitis. *Laryngosc. Investig. Otol.* **5**, 992–1002 (2020). 10.1002/lio2.485 PMC7752087 33364387

[27] Hummel, T. & Frasnelli, J. The intranasal trigeminal system. in *Handbook of Clinical Neurology* vol. 164 119–134 (Elsevier, (2019). 10.1016/B978-0-444-63855-7.00008-3 31604542

[28] Migneault-Bouchard, C., Hsieh, J. W., Hugentobler, M., Frasnelli, J. & Landis, B. N. Chemosensory decrease in different forms of olfactory dysfunction. *J. Neurol.* **267**, 138–143 (2020). 31586261 10.1007/s00415-019-09564-x

[29] Scheibe, M., Schulze, S., Mueller, C. A., Schuster, B. & Hummel, T. Intranasal trigeminal sensitivity: measurements before and after nasal surgery. *Eur. Arch. Otorhinolaryngol.* **271**, 87–92 (2014). 23568039 10.1007/s00405-013-2466-4

[30] Hernandez, A. K., Uhl, C., Haehner, A., Cuevas, M. & Hummel, T. Objective nasal airflow measures in relation to subjective nasal obstruction, trigeminal function, and olfaction in patients with chronic rhinosinusitis. *Rhin* (2024). 10.4193/Rhin23.270 38507726

[31] Hu, B. et al. Olfactory event-related potential in patients with rhinosinusitis-induced olfactory dysfunction. *Am. J. Rhinol. Allergy* **24**, 330–335 (2010). 21244732 10.2500/ajra.2010.24.3517

[32] Lund, V. J. & Mackay, I. S. Staging in rhinosinusitus. *Rhinology* **31**, 183–184 (1993). 8140385

[33] Widal, F., Abrami, P., Lermoyez, J., Widal, F. & Abrami, P. First complete description of the aspirin idiosyncrasy-asthma-nasal polyposis syndrome (plus urticaria)--1922 (with a note on aspirin desensitization). By J. Lermoyez. *J. Asthma* **24**, 297–300 (1987). 3327855

[34] Baumann, I., Blumenstock, G., DeMaddalena, H., Piccirillo, J. F. & Plinkert, P. K. Lebensqualität bei Patienten mit chronischer Rhinosinusitis: Validierung des Sino-Nasal Outcome Test-20 German Adapted Version. *HNO* **55**, 42–47 (2007). 16328203 10.1007/s00106-005-1347-6

[35] Piccirillo, J. F., Merritt, M. G. J. & Richards, M. L. Psychometric and clinimetric validity of the 20-Item Sino-Nasal Outcome Test (SNOT-20). *Otolaryngol. Head Neck Surg.* **126**, 41–47 (2002). 11821764 10.1067/mhn.2002.121022

[36] Ottaviano, G. & Fokkens, W. J. Measurements of nasal airflow and patency: a critical review with emphasis on the use of peak nasal inspiratory flow in daily practice. *Allergy* **71**, 162–174 (2016). 26447365 10.1111/all.12778

[37] Hummel, T., Sekinger, B., Wolf, S. R., Pauli, E. & Kobal, G. Sniffin’ sticks’: olfactory performance assessed by the combined testing of odor identification, odor discrimination and olfactory threshold. *Chem. Senses*. **22**, 39–52 (1997). 9056084 10.1093/chemse/22.1.39

[38] Oleszkiewicz, A., Schriever, V. A., Croy, I., Hähner, A. & Hummel, T. Updated Sniffin’ Sticks normative data based on an extended sample of 9139 subjects. *Eur. Arch. Otorhinolaryngol.* **276**, 719–728 (2019). 30554358 10.1007/s00405-018-5248-1 PMC6411676

[39] Kobal, G. & Hummel, C. Cerebral chemosensory evoked potentials elicited by chemical stimulation of the human olfactory and respiratory nasal mucosa. *Electroencephalogr. Clin. Neurophysiol.* **71**, 241–250 (1988). 2454788 10.1016/0168-5597(88)90023-8

[40] Kobal, G. & Plattig, K. H. [Objective olfactometry: methodological annotations for recording olfactory EEG-responses from the awake human]. *EEG EMG Z. Elektroenzephalogr Elektromyogr Verwandte Geb*. **9**, 135–145 (1978). 100308

[41] Hummel, T. & Kobal, G. Olfactory event-related potentials. In Methods in Chemosensory Research (ed. Simon S.A., Nicolelis M. A. L.) 429–464 (Boca Raton: CRC Press 2001).

[42] Hummel, T. et al. Chemosensorisch evozierte Potentiale zur klinischen Diagnostik von Riechstörungen. *HNO* **48**, 481–485 (2000). 10929232 10.1007/s001060050602

[43] Iravani, B., Arshamian, A., Ohla, K., Wilson, D. A. & Lundström, J. N. Non-invasive recording from the human olfactory bulb. *Nat. Commun.* **11**, 648 (2020). 32005822 10.1038/s41467-020-14520-9 PMC6994520

[44] Jasper, H. H. Report of the committee on methods of clinical examination in electroencephalography. *Electroencephalogr. Clin. Neurophysiol.* **10**, 370–375 (1958).

[45] Mignot, C. et al. Migraine with aura: less control over pain and fragrances? *J. Headache Pain*. **24**, 55 (2023). 37198532 10.1186/s10194-023-01592-3 PMC10189721

[46] Rombaux, P., Mouraux, A., Bertrand, B., Guerit, J. & Hummel, T. Assessment of olfactory and trigeminal function using chemosensory event-related potentials. *Neurophysiologie Clinique/Clinical Neurophysiol.* **36**, 53–62 (2006). 10.1016/j.neucli.2006.03.005 16844543

[47] Gallo, S. et al. Prognostic value of the Sinonasal Outcome Test 22 (SNOT-22) in chronic rhinosinusitis. *Acta Otorhinolaryngol. Ital.* **40**, 113–121 (2020). 32469005 10.14639/0392-100X-N0364 PMC7256904

[48] Guo, Y. et al. Outcomes of endoscopic sinus surgery in patients with central compartment atopic disease. *World Allergy Organ. J.* **17**, 100859 (2024). 38312493 10.1016/j.waojou.2023.100859 PMC10837641

[49] Saratziotis, A. et al. Endoscopic sinus surgery outcomes in CRS: quality of life and correlations with NOSE scale in a prospective cohort study. *Eur. Arch. Otorhinolaryngol.* **278**, 1059–1066 (2021). 32897442 10.1007/s00405-020-06334-8

[50] Tashman, K. et al. Five-year EuroQol 5-dimension outcomes after endoscopic sinus surgery. *Laryngoscope* **134**, 2592–2601 (2024). 38126531 10.1002/lary.31206

[51] Hopkins, C., Rudmik, L. & Lund, V. J. The predictive value of the preoperative Sinonasal Outcome Test-22 score in patients undergoing endoscopic sinus surgery for chronic rhinosinusitis. *Laryngoscope* **125**, 1779–1784 (2015). 25891944 10.1002/lary.25318

[52] Soler, Z. M. et al. Sino-nasal outcome test‐22 outcomes after sinus surgery: A systematic review and meta‐analysis. *Laryngoscope* **128**, 581–592 (2018). 29164622 10.1002/lary.27008 PMC5814358

[53] Katotomichelakis, M. et al. Predictors of quality of life outcomes in chronic rhinosinusitis after sinus surgery. *Eur. Arch. Otorhinolaryngol.* **271**, 733–741 (2014). 23842603 10.1007/s00405-013-2626-6

[54] Smith, T. L. et al. Long-term outcomes of endoscopic sinus surgery in the management of adult chronic rhinosinusitis. *Int. Forum Allergy Rhinol.* **9**, 831–841 (2019). 31207172 10.1002/alr.22369 PMC6685750

[55] Hernandez, A. K. et al. Predictors of olfactory improvement after endoscopic sinus surgery in chronic rhinosinusitis with nasal polyps. *J. Laryngol. Otol.* **137**, 524–531 (2023). 35791849 10.1017/S0022215122001633

[56] Lötsch, J. & Hummel, T. Clinical usefulness of self-rated olfactory performance-a data science-based assessment of 6000 patients. *Chem. Senses* **44**, 357–364 (2019). 31077277 10.1093/chemse/bjz029

[57] Brämerson, A. et al. Event-related potentials in patients with olfactory loss. *Acta Otolaryngol.* **128**, 1126–1131 (2008). 18607946 10.1080/00016480801891702

[58] Burghardt, G. K. L., Cuevas, M., Sekine, R. & Hummel, T. Trigeminal sensitivity in patients with allergic rhinitis and chronic rhinosinusitis. *Laryngoscope* **133**, 654–660 (2023). 36504410 10.1002/lary.30512

[59] Lötsch, J. & Hummel, T. The clinical significance of electrophysiological measures of olfactory function. *Behav. Brain Res.* **170**, 78–83 (2006). 16563529 10.1016/j.bbr.2006.02.013

[60] Rombaux, P., Bertrand, B., Keller, T. & Mouraux, A. Clinical significance of olfactory event-related potentials related to orthonasal and retronasal olfactory testing. *Laryngoscope* **117**, 1096–1101 (2007). 17460578 10.1097/MLG.0b013e31804d1d0d

[61] Song, J., Wang, M., Wang, C. & Zhang, L. Olfactory dysfunction in chronic rhinosinusitis: insights into the underlying mechanisms and treatments. *Expert Rev. Clin. Immunol.* **19**, 993–1004 (2023). 37432663 10.1080/1744666X.2023.2235891

[62] Zhang, L. et al. Correlation of tissue eosinophil count and chemosensory functions in patients with chronic rhinosinusitis with nasal polyps after endoscopic sinus surgery. *Eur. Arch. Otorhinolaryngol.* **276**, 1987–1994 (2019). 30937558 10.1007/s00405-019-05413-9

[63] Poletti, S. C., Cuevas, M., Weile, S. & Hummel, T. Trigeminal sensitivity in chronic rhinosinusitis: topographical differences and the effect of surgery. *Rhinology* **55**, 70–74 (2017). 28026837 10.4193/Rhin16.194

[64] Vavrina, J. J., Hummel, T., Landis, B. N., Macario, S. & Soyka, M. B. Intranasal trigeminal and secretory functions are impaired after topical anaesthesia or surgical treatment of epistaxis. *Rhinology* **63**, 608–615 (2025). 40657797 10.4193/Rhin24.314

[65] Whitcroft, K. L., Noltus, J., Andrews, P. & Hummel, T. Sinonasal surgery alters brain structure and function: Neuroanatomical correlates of olfactory dysfunction. *J. Neurosci. Res.* **99**, 2156–2171 (2021). 34110641 10.1002/jnr.24897

[66] Whitcroft, K. L. et al. Structural plasticity of the primary and secondary olfactory cortices: increased gray matter volume following surgical treatment for chronic rhinosinusitis. *Neuroscience* **395**, 22–34 (2018). 30326289 10.1016/j.neuroscience.2018.10.011

[67] Liu, D. T. et al. Factors associated with relevant olfactory recovery after olfactory training: a retrospective study including 601 participants. *Rhinology* **59**, 91–97 (2021). 33544097 10.4193/Rhin20-262

