# Supplementary material for: Effects of functional endoscopic sinus surgery on olfactory and trigeminal function in chronic rhinosinusitis with nasal polyps
Source: Sci Rep. 2026 Jul 7;16:20984. doi: 10.1038/s41598-026-58587-8 (PMC13341755; doi:10.1038/s41598-026-58587-8)
Supplement: Supplementary file 1 — Supplementary Material 1 [file 41598_2026_58587_MOESM1_ESM.docx]

**EFFECTS OF FUNCTIONAL ENDOSCOPIC SINUS SURGERY ON OLFACTORY AND TRIGEMINAL FUNCTION IN CHRONIC RHINOSINUSITIS WITH NASAL POLYPS**

**Authors**

Arianna Soncini^2^, Kwangsu Kim^1^, Fabian Herfort^1^, Coralie Mignot^1^, Antje Haehner^1^, Thomas Hummel^1^

**Affiliations**

1 Smell and Taste Clinic, Department of Otorhinolaryngology, University of Dresden Medical School, Technische Universität Dresden, Fetscherstrasse 74, 01307 Dresden, Germany

2 Department of Medicine and Surgery, University of Parma, via Gramsci 14, 43121, Parma, Italy

**Corresponding Author**

Kwangsu Kim, PhD

Smell and Taste Clinic, Department of Otorhinolaryngology, University of Dresden Medical School, Technische Universität Dresden, Fetscherstrasse 74, 01307 Dresden, Germany

Telephone number: +4915773808175

E-mail address: kwangsu.kim@tu-dresden.de

**Supplementary Information (SI)**

| Outcome Measure | Age  (p-value) | Age × Group Interaction  (p-value) |
| --- | --- | --- |
| SOLF | 0.684 | 0.840 |
| SNF | 0.817 | 0.842 |
| SNOT-20 | 0.110 | 0.592 |
| PNIF | 0.699 | 0.960 |
| TDI | 0.185 | 0.669 |
| PEA Intensity | 0.165 | 0.298 |
| PEA Pleasantness | 0.690 | 0.183 |
| CO₂ Intensity | 0.202 | 0.255 |
| CO₂ Pleasantness | 0.067 | 0.480 |
| PEA Cz N1 Latency | 0.807 | 0.379 |
| PEA Cz P2 Latency | 0.191 | 0.489 |
| PEA Cz N1–P2 Amplitude | 0.612 | 0.775 |
| CO₂ Cz N1 Latency | 0.544 | 0.334 |
| CO₂ Cz P2 Latency | 0.297 | 0.334 |
| CO₂ Cz N1–P2 Amplitude | 0.967 | 0.189 |

**Supplementary Table S1** **Influence of age on subjective, psychophysical, chemosensory, and electrophysiological outcome measures.** Additional repeated-measures ANOVA were performed including age as a covariate. Homogeneity of regression slopes was assessed by testing the Age × Group interaction for each outcome measure. No significant Age × Group interactions were observed (all p > 0.05).
